# Efficacy of Web-Based Weight Loss Maintenance Programs: A Randomized Controlled Trial Comparing Standard Features Versus the Addition of Enhanced Personalized Feedback over 12 Months

**DOI:** 10.3390/bs7040076

**Published:** 2017-11-08

**Authors:** Clare E. Collins, Philip J. Morgan, Melinda J. Hutchesson, Christopher Oldmeadow, Daniel Barker, Robin Callister

**Affiliations:** 1 Nutrition and Dietetics, School of Health Sciences, Faculty of Health and Medicine, The University of Newcastle, Callaghan, NSW 2308, Australia; Melinda.Hutchesson@newcastle.edu.au; 2 Priority Research Centre in Physical Activity and Nutrition, The University of Newcastle, Callaghan, NSW 2308, Australia; Philip.Morgan@newcastle.edu.au (P.J.M.); Robin.Callister@newcastle.edu.au (R.C.); 3 School of Education, Faculty of Education and Arts, The University of Newcastle, Callaghan, NSW 2308, Australia; 4 Clinical Research Design, IT and Statistical Support Unit, Hunter Medical Research Institute, Level 3 Pod, HMRI building Lot 1, Kookaburra Circuit, New Lambton Heights, NSW 2305, Australia; Christopher.Oldmeadow@hmri.org.au (C.O.); Daniel.Barker@newcastle.edu.au (D.B.); 5 School of Medicine and Public Health, Faculty of Health and Medicine, The University of Newcastle, Callaghan, NSW 2308, Australia; 6 School of Biomedical Sciences and Pharmacy, Faculty of Health and Medicine, The University of Newcastle, Callaghan, NSW 2308, Australia

**Keywords:** intervention, weight loss, web-based, randomized controlled trial, calorie restriction, eHealth

## Abstract

Few randomized controlled trials (RCT) have evaluated the efficacy of web-based programs targeting maintenance of lost weight. The aims of this study were to evaluate two versions of a commercially available web-based weight loss maintenance (WLM) program and examine whether the provision of enhanced feedback was associated with better WLM. The study was an assessor-blinded RCT of change in body mass index (BMI) over 12 months WLM. Participants were 227 adults (44% male, 42.3 ± 10.1 years, BMI 30.4 ± 4.1 kg/m^2^) randomized to either a basic (Basic WLM) or enhanced program with additional support (Enhanced WLM). Analysis was intention-to-treat with imputation using last observation carried forward. There was no significant weight rebound from the start of weight loss maintenance to 12 months for either group (mean: basic 1.3%, enhanced 1.5%) and limited change in secondary outcomes for either program. There were no significant between-group differences in the primary outcome of change in BMI (basic −0.5 (1.9) kg/m^2^, enhanced −0.5 (1.6) kg/m^2^, *p* = 0.93). In conclusion, a web-based WLM program was effective in preventing weight regain over one year following weight loss. However, the addition of personalized e-feedback provided limited additional benefits compared to a standard program. Given the potential reach of web-based approaches, further research examining which web-based program components optimize weight outcomes long-term is required.

## 1. Introduction

Obesity rates are continuing to rise globally [1] in contrast to the limited access to treatment programs. While web-based approaches to treatment could potentially have broad reach, especially as households gain access to broadband internet, their evaluation in the context of longer-term follow-up has been limited.

Studies have shown that passive follow-up with no active intervention after weight loss is associated with weight gain [2], with 50–80% of participants gradually regaining weight lost following treatment, with most regain occurring in the first year [3,4,5,6]. A systematic review of weight loss maintenance (WLM) trials found that active WLM interventions facilitated a 1.56 kg (95% CI −2.27 to −0.86 kg) lower weight regain compared with passive follow-up after 12 months. Most WLM studies have required attendance at group sessions and have been hampered by methodological issues, such as mainly recruiting females or mid-aged adults and including only those with a good response to an initial weight loss phase, while excluding those with low adherence. [7]. Few studies have evaluated the use of eHealth technologies (e.g., websites, smartphone applications, text messages) to deliver WLM interventions. Furthermore, most research in this area has looked at short-term interventions (<6 months) without longer-term follow-up [8,9]. The small number of longer-term studies have reported a gradual regain of weight lost following treatment, with regain occurring after the first six months [10,11]. A recent randomized controlled trial examined the effectiveness of a 12-week short message service (SMS) based WLM program following an initial 12-week commercial weight loss program. At three and nine months follow-up, there was no significant difference in weight maintenance between the usual care control group who received a brief telephone call providing lifestyle information and a mailed leaflet with advice on weight loss maintenance and the intervention group who received a weekly SMS to remind them to self-weigh in addition to the call [12].

A systematic review published in 2017, evaluated the efficacy of web-based interventions on weight loss or WLM in adults with overweight or obesity and found web-based interventions were more effective than minimal treatments but less effective than face-to-face interventions [13]. Furthermore, a recent systematic review concluded that there was insufficient evidence to recommend the use of eHealth interventions for WLM [14]. There were however promising results from the eHealth weight loss trials with the addition of newer technologies including text messages, self-monitoring devices, and mobile applications, with meta-analysis demonstrating significantly greater weight loss (1.46 kg [0.80, 2.13], *p* < 0.001) in interventions with enhanced behavioral features (e.g., individualized counselling, feedback on dietary intake or weight change) or technologies (e.g., addition of text messaging) than standard eHealth interventions.

There is some evidence to suggest that these ‘enhanced’ interventions positively impact participant engagement, retention, and behavior change. We have previously published results of an online commercially available weight loss maintenance (WLM) program which demonstrated limited between-group differences for the basic and enhanced versions of the program after six months [15].

While the enhanced version resulted in greater retention (81.0% vs. 68.5%) and usage of the web-program, it did not result in a difference in weight loss [15].

A study using a 12-month intervention program (6-month weight loss plus a 6-month WLM) compared a self-directed web-based commercial program to a therapist-led behavioral web-based program and found greater weight loss in the behavioral web-based program than the therapist-led program at 12 months [16]. We have previously reported that adults randomized to this therapist-led web-based weight loss program initially lost more weight compared to wait list controls [17], and that there was no difference in weight loss between basic or enhanced versions of the weight loss program after either 12 weeks [17] or after 24 weeks [15] of participation.

To date, no WLM trials have specifically evaluated the inclusion of enhanced behavioral features in an eHealth WLM program, including those outlined in Table S1, such as automatically-generated personalized reports; personalized feedback on diet, physical activity, and weight loss, as well as reminders to use the online diary, visit the website, and weigh-in.

Therefore, the aim of the current study was to evaluate the impact of two versions of a WLM program on BMI in adults with overweight or obesity who had previously completed a weight loss program. The secondary aim was to compare differences in waist circumference and clinical measures including cholesterol, triglycerides, glucose and insulin between a standard version of a web-based WLM program (basic) and an enhanced version that provided additional personalized e-support.

## 2. Materials and Methods

### 2.1. Participants

Adults (BMI 25–40 kg/m^2^), aged 18–60 years were initially recruited into a weight loss trial in 2009 from the Hunter community in NSW, Australia [18]. Written informed consent was obtained from all participants for the weight loss maintenance trial. The study was approved by the University of Newcastle Human Ethics Research Committee (H-2009-0245) on 10 September 2009. The trial was registered with the Australian New Zealand Clinical Trials Registry, anzctr.org.au ACTRN12610000197033.

### 2.2. Study Design

**Figure 1:**
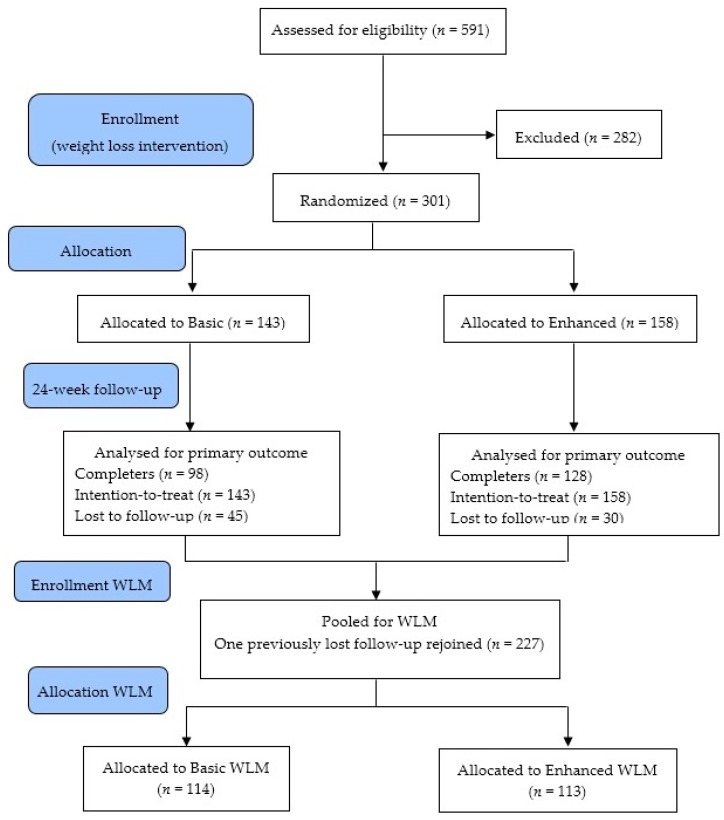
Participant flow from baseline of initial 24-week web-based weight loss intervention to group allocation in the WLM program. Further detail regarding participant flow has been published [15].

Participants who completed an initial 24-week web-based weight loss intervention (The Biggest Loser Club, SP Health Co Pty Ltd., North Sydney, Australia) were randomly assigned into one of two weight loss maintenance groups for 12 months using a stratified randomized block design. Participants were allocated to either a basic weight loss maintenance program (Basic WLM) or an enhanced version of the weight loss maintenance program (Enhanced WLM) (see Figure 1). Both participants and the outcome assessors were blinded to group allocation. Detailed methods of the RCT have been published elsewhere [18]. The WLM programs were developed to follow on directly after completion of 24 weeks of a web-based weight loss program [15]. Program features and differences between groups are summarized in Table S1 and also described in detail elsewhere [18]. The Basic WLM group received free access to the weight loss program, but the weekly meal plans were based on an energy intake target equivalent to weight maintenance. The Enhanced WLM group received access to these same meal plans and dietary information, but they also received personalized system-generated feedback on their progress. The feedback on progress was provided using system-generated personalized reports that were populated with the data entered into the platform by the participant. Feedback included progress in relation to weight maintenance goals; usage of the online diet and physical activity diary (which was recommended but not mandated); usage patterns for website features; and level of success with weight loss. Those allocated to the enhanced group also received an escalating scale of email reminders followed by SMS text message reminders (if no response) to use the diary, visit the website, to ‘weigh-in’ by recording their weekly weight in the online program, and a relapse phone call if their recorded weight rebounded by ≥3% to remind them to return to weight loss mode (Table S1).

### 2.3. Measures

Participants were assessed at baseline and 12 months [18] by blinded research assistants. Height, weight, waist circumference, and blood pressure were measured using standardized procedures [18] and BMI calculated. Following an overnight fast, blood samples were collected and analyzed for total cholesterol, low density lipoprotein (LDL), high density lipoprotein (HDL) cholesterol, triglycerides, glucose, and insulin by a single National Association of Testing Authorities accredited pathology service using standard automated techniques.

### 2.4. Data Analysis

Baseline variables were compared between treatment groups using analysis of variance (ANOVA) for continuous variables and chi-square tests for categorical variables. Analysis of covariance (ANCOVA) was used to test for group differences in outcomes at 12 months after adjusting for the baseline values of the outcome and sex. Analyses were performed in Stata v11 or SAS v9.2 (StataCorp, College Station, TX, USA). Intention-to-treat (ITT) analyses included all participants randomized at the weight loss management phase baseline, with missing follow-up data imputed using the last observation carried forward (LOCF).

## 3. Results

At entry into WLM 227 participants were randomized to Basic WLM (*n* = 114) or Enhanced WLM (*n* = 113) program versions, with demographic data summarized in Table 1. Mean age was 43.3 (±9.7) years, with a mean BMI of 30.6 (±4.1) kg/m^2^, 42% were male and 95% were Australian-born (Table 1). There was no significant between-group difference in attrition after 12 months, basic 18.4% and enhanced 23.9%, *p* = 0.31.

Both the Basic and Enhanced WLM groups successfully achieved WLM after 12 months with no significant rebound in weight. There were no significant between-group differences (*p* > 0.05) for the primary outcome of BMI with mean weight change (12 months—baseline) similar between the basic (−0.5 (1.9) kg/m^2^) and enhanced groups (−0.5 (1.6) kg/m^2^), *p* > 0.05 (Table 2). Table 2 shows that there were no significant differences in secondary outcomes between groups up to 12 months (*p* > 0.05).

## 4. Discussion

Whilst participants in both intervention arms of the current trial successfully maintained weight over one year of participating in a web-based program design to support maintenance of lost weight, those with access to the additional features designed to provide support and personalized feedback on diet, physical activity, and weight loss did not achieve better outcomes compared to those receiving access to the online program with minimal support. Both groups regained approximately one-and-a-half kilograms over the 12-month maintenance period, which was not significantly different between groups. This is comparable to results of the active intervention arms from a recent systematic review of all WLM intervention modes that included both food intake and physical activity advice, including group programs, face-to-face, and internet interventions [7]. The review included almost 3000 participants across 25 comparisons and found that the active intervention arms were associated with less weight regain of approximately 1.56 kg, up to 12 months [7]. This suggests that both active intervention arms in the current study were successful in achieving WLM. In our study, it appears that doing ‘anything’ was enough to support WLM, compared to the no-intervention arms in the systematic review, which also found no evidence that specific modes of intervention delivery were more effective than others. This suggests that having access to the level support provided in both the basic and enhanced versions of the current web-based WLM intervention, following a weight loss intervention did facilitate maintenance of lost weight. Given that it is well established that weight regain is common following initial weight loss [19], the results of the current study are important.

Our systematic review evaluating effectiveness of 56 interventions that included a specific dietary component within the WLM intervention found that 14 achieved significant between-group differences at follow-up [2]. Furthermore, our recent systematic review and meta-analysis which evaluated the effectiveness of 84 e-Health interventions, found that e-Health weight loss interventions that include extra support strategies including counselling, personalized feedback, motivational interviewing and/or personal contact, appear to achieve significantly greater weight loss compared with standard eHealth interventions [13]. However, five studies which focused specifically on WLM interventions found no significant difference in weight change between eHealth WLM programs vs. control [14]. The authors concluded that there is currently insufficient evidence to recommended eHealth interventions for WLM and that further high-quality research is required to determine their effectiveness [14]. Although the included studies were heterogeneous the typical intervention characteristics were WLM interventions that lasted 39 weeks on average, had approximately 180 participants and attrition of 26% compared to the current WLM study which lasted 52 weeks had 227 participants and an attrition rate of 21%.

The current study is important because individual studies to date suggest that face-to-face contact involving either weekly or monthly individual or group counseling sessions are more likely to achieve WLM than internet support in studies lasting 18 to 30 months. Recent systematic reviews on WLM interventions [7,14] suggest that there is a need for extended support for weight management following participation in weight loss programs internationally, as in-person attendance is not likely to be viable long-term. Hence, prolonged WLM support using eHealth technologies need to be evaluated for both feasibility, engagement, and cost-effectiveness in the long-term.

Limitations include the lack of a wait list control group, which is similar to the majority of WLM maintenance studies to date [2]. Although the small sample size and attrition likely reduced the power to detect significant differences between groups for the secondary outcomes, it is similar to other WLM trials lasting 12 or more months which had an average drop-out of 20% [7]. Furthermore. results from the current study need to be interpreted with caution given the meta-analysis within the systematic review [14] indicates that there is no significant difference between the support methods during WLM. This is also supported by our review of web-based interventions which also reported no difference between these two methods of delivery [20]. Strengths include the RCT design, use of blinded assessors, and the comparison of two versions of the WLM program for 12 months, following an initial period of weight loss. Future studies should consider evaluating cost-effectiveness and efficacy in specific population groups for whom access services may be a challenge, based on a range of issues related to time, cost, convenience, rurality, health conditions, or other socio-economic factors.

In conclusion, a commercial web-based weight loss maintenance program, with a specific intervention component targeting maintenance of lost weight can be effective at preventing weight re-gain up to one year following 24 weeks of weight loss. While the addition of enhanced features that provide additional feedback and social support did not provide additional benefits during maintenance, health professionals should advise clients that a specific WLM strategy does facilitate WLM up to one year following weight loss. Further research addressing level of feedback and support required to optimize weight status long-term in an online environment is required.

## Figures and Tables

**Table 1:** Demographic and other baseline characteristics of those randomized to 12 months of either a basic or enhanced features weight loss maintenance (WLM) web-based program.

Characteristic	Subgroup or Mean (SD)	Treatment Group			*p*-Value
		Basic WLM (*n* = 114)	Enhanced WLM (*n* = 113)	All (*n* = 227)	
Sex	Men	48 (42%)	52 (46%)	100 (44%)	0.55
Country of birth	Australia	108 (95%)	100 (88%)	208 (92%)	0.23
Highest level of education	School	37 (32%)	28 (25%)	65 (29%)	0.49
	Trade/Diploma	41 (36%)	40 (35%)	81 (35%)	
	University Degree	24 (21%)	28 (25%)	52 (23%)	
	Higher University Degree	12 (11%)	17 (15%)	29 (13%)	
Weekly household income (AUS$)	<$700	12 (11%)	7 (6.4%)	19 (8.8%)	0.60
	$700 to <$1000	6 (5.7%)	5 (4.5%)	11 (5.1%)	
	$1000 to <$1400	13 (12%)	11 (10%)	24 (11%)	
	$1500 or more	71 (67%)	84 (76%)	155 (72%)	
Age (years)	mean (SD)	43.3 (9.7)	41.3 (10.3)	42.3 (10.1)	0.14
Height (m)	mean (SD)	1.70 (0.09)	1.72 (0.09)	1.71 (0.09)	0.17
Weight (kg)	mean (SD)	88.5 (14.5)	89.3 (15.6)	88.9 (15.1)	0.70
Body mass index (kg/m^2^)	mean (SD)	30.6 (4.1)	30.1 (4.1)	30.4 (4.1)	0.45
Waist circumference at umbilicus (cm)	mean (SD)	100.6 (11.1)	98.5 (11.7)	99.6 (11.4)	0.18
Waist circumference at narrowest point (cm)	mean (SD)	93.2 (11.5)	92.2 (11.9)	92.7 (11.7)	0.52
Waist to height ratio at umbilicus	mean (SD)	0.59 (0.07)	0.57 (0.07)	0.58 (0.07)	0.04
Waist to height ratio at narrowest point	mean (SD)	0.55 (0.07)	0.54 (0.06)	0.54 (0.06)	0.15
Weight loss at baseline entry to study (24 weeks)	mean (SD)	−4.38 (5.30)	−4.47 (6.37)	−4.42 (5.84)	0.91
Systolic blood pressure (mmHg)	mean (SD)	117.5 (13)	117.7 (12)	117.6 (12.5)	0.92
Diastolic blood pressure (mmHg)	mean (SD)	77.6 (10.6)	77.2 (9.2)	77.4 (9)	0.76
Resting heart rate (bpm)	mean (SD)	64.6 (9.5)	64.7 (10.2)	64.6 (9.8)	0.94
Total serum cholesterol (mmol/L)	mean (SD)	5.2 (1.1)	4.8(1.0)	5.0 (1.1)	0.03
LDL cholesterol (mmol/L)	mean (SD)	3.1 (1.0)	2.9 (0.8)	3.0 (0.9)	0.08
HDL cholesterol (mmol/L)	mean (SD)	1.3 (0.3)	1.3 (0.3)	1.3 (0.3)	0.41
Triglycerides (mmol/L)	mean (SD)	1.5 (1.0)	1.3 (0.7)	1.4 (0.9)	0.16
Glucose (mmol/L)	mean (SD)	4.6 (0.6)	4.5 (0.7)	4.6 (0.7)	0.08
Insulin (mIU/L)	mean (SD)	6.6 (5.5)	6.9 (6.2)	6.7 (5.9)	0.73

**Table 2:** Mean (SD) change in a anthropometric and clinical variables from WLM baseline to 12-month follow-up within each treatment group and least square mean (LSM) difference (95% CI) in change between treatments groups (Completed the six month Baseline, ITT/LOCF approach).

Characteristic	Treatment Group Mean Change (SD)		Absolute Difference between Groups LSM (95% CI)	*p* Values for Group Effect
	Basic	Enhanced	Enhanced vs. Basic	Difference at Specified Time
Weight (kg)	1.3 (5.1)	1.5 (4.4)	0.15 (−1.09, 1.39)	0.81
Systolic blood pressure (mmHg)	1.1 (12.1)	1.1 (10.2)	0.24 (−2.59, 3.08)	0.87
Diastolic blood pressure (mmHg)	−1.5 (8.1)	−0.1 (6.5)	1.15 (−0.76, 3.06)	0.24
Body mass index (kg/m^2^)	0.5 (1.9)	0.5 (1.6)	0.02 (−0.46, 0.50)	0.93
Pulse rate (bpm)	−0.3 (6.7)	0.5 (5.8)	0.74 (−0.88, 2.35)	0.37
Waist circumference at umbilicus (cm)	3.8 (5.4)	3.3 (4.9)	0.76 (−0.63, 2.16)	0.28
Waist circumference at narrowest point (cm)	2.6 (4.6)	2.6 (4.6)	0.11 (−1.12, 1.35)	0.86
Waist to height ratio at umbilicus	0.0 (0.0)	0.0 (0.0)	0.01 (−0.00, 0.01)	0.21
Waist to height ratio at narrowest point	0.0 (0.0)	0.0 (0.0)	0.00 (−0.01, 0.01)	0.80
Total serum cholesterol (mmol/L)	0.1 (0.8)	0.2 (0.7)	0.07 (−0.12, 0.26)	0.50
LDL cholesterol (mmol/L)	0.0 (0.6)	0.1 (0.5)	0.01 (−0.15, 0.16)	0.94
HDL cholesterol (mmol/L)	0.0 (0.2)	0.0 (0.2)	0.00 (−0.06, 0.06)	0.88
Triglycerides (mmol/L)	0.0 (0.5)	0.2 (0.5)	0.13 (−0.02, 0.27)	0.10
Glucose (mmol/L)	0.1 (0.5)	0.2 (0.5)	0.08 (−0.05, 0.22)	0.20
Insulin (mIU/L)	1.6 (4.2)	0.89 (6.5)	0.61 (−0.719, 2.01)	0.39

## Supplementary Materials

The following are available online at www.mdpi.com/2076-328X/7/4/76/s1, Table S1: Description of the basic and enhanced commercial web-based weight loss maintenance programs.
