# Supplementary material for: Efficacy of Web-Based Weight Loss Maintenance Programs: A Randomized Controlled Trial Comparing Standard Features Versus the Addition of Enhanced Personalized Feedback over 12 Months
Source: Behav Sci (Basel). 2017 Nov 8;7(4):76. doi: 10.3390/bs7040076 (PMC5746685; doi:10.3390/bs7040076)
Supplement: Supplementary file 1 [file behavsci-07-00076-s001.pdf]

Table S 1 Description of the basic and enhanced commercial web-based weight loss maintenance programs

Basic and Enhanced	Additional features in Enhanced only
**Diet and exercise recommendations**	
• Participants given instructions on how to adjust calorie target to maintain weight loss.<br>• Individualized daily calorie targets to facilitate weight maintenance, or 0.5-1kg weight loss per week (~ 2600kJ less than their estimated energy requirements) if continuing to lose weight.<br>• Access to weekly low-fat menu plan and grocery lists designed to meet nutrient reference values and assigned calorie target.	• Personalized automated maintenance mentor reports suggesting appropriate weight maintenance goals and key behavior changes required for success. Eating behaviors targeted included total energy, saturated fat and fiber intake, daily serves of fruit and vegetables, "high-risk eating behaviors" (e.g. skipping meals, not eating breakfast, drinking soft drinks) and non-hungry eating triggers.
**Self-monitoring**	
• Web-based food and exercise diary to monitor energy intake and energy expenditure. Daily and weekly calculations of energy balance and detailed nutrition summaries compared to recommended nutrient targets if food entries made in online diary.<br>• Participants set calorie targets to maintain weight loss, advised to self-monitor their weight, waist and hip girths.<br>• Encouraged to self-monitor via weekly e-mail and/or short message service (SMS) reminders to enter weight on website. Entered data tracked and displayed graphically.	• Eating behaviors targeted consistent with personal enrolment reports.<br>• Reminders to self-monitor using online diary and/or record weekly weight. The reminder schedule included an initial reminder email; if no response, a text message; if no response, a phone call for weigh-ins only.

**Education materials**	
• Online education in the form of weekly tutorials, fact sheets, meal, and exercise plans and weekly challenges with a focus specifically on maintenance of lost weight.	• Reminders to visit the website
**Feedback**	
• Automatic weight relapse program if participant gained more than 3% of initial body weight. Participant moved back into weight loss mode and received weight loss calorie target with appropriate strategies to get back to weight maintenance goal. Once achieved, participant cycled back into weight maintenance.	• Weekly and monthly automated personalized feedback for key elements of diet and physical activity based on diary entries; usage patterns for website features; and level of success with weight loss.
**Social Support**	
• Social support via online discussion forums	• Initial phone call by dietitian to introduce the maintenance program features<br>• Weight relapse phone call to discuss strategies to achieve weight maintenance goal
